# Periodontitis is associated with insulin resistance in adults living with diabetes mellitus in Uganda: a cross- sectional study

**DOI:** 10.1186/s13104-023-06473-1

**Published:** 2023-09-14

**Authors:** Haruna Muhmood Kiryowa, Ian Guyton Munabi, William Buwembo, Charles Mugisha Rwenyonyi, Erisa Sabakaki Mwaka, Mark Kaddumukasa

**Affiliations:** 1 Department of Anatomy, Makerere University College of Health Sciences, P.O. Box 7072, Kampala, Uganda; 2 School of Dentistry, Makerere University College of Health Sciences, P.O. Box 7072, Kampala, Uganda; 3 Department of Medicine, Makerere University College of Health Sciences, P.O. Box 7072, Kampala, Uganda

**Keywords:** Diabetes mellitus, Insulin resistance, Periodontitis and triglyceride glucose index

## Abstract

**Introduction:**

Evidence suggests that majority of patients with diabetes mellitus in Uganda have poor glycaemic control as well as periodontal disease. This study set out to determine the association between periodontitis and insulin resistance in adult patients with diabetes mellitus in Uganda using the triglyceride glucose index.

**Methods:**

Two hundred and twenty-three adult study participants with confirmed diabetes mellitus were enrolled in a cross-sectional study. Oral examination was carried with the aid of a periodontal probe to determine the periodontal status and findings recorded using the WHO Oral Health Assessment Tool for Adults, 2013. We recorded clinical details for body mass index (BMI in kg/m^2^) and laboratory parameters including fasting blood sugar (mmol/L), glycated hemoglobin levels (HbA1c, %) and serum triglycerides (mmol/L) using a study questionnaire. Data were analyzed using R version 4.10. The glucose triglyceride index was used as a measure of insulin resistance. Logistic regression analysis carried out to determine the factors associated insulin resistance.

**Results:**

The majority of the study participants was female (70%) with an average age of 48.5 years (SD+/- 11.1). The mean body mass index was 29.6 kg/m^2^ (SD+/- 5.82). The mean serum triglyceride index was 9.48 (SD+/- 0.675). Eighty-six-point 1% of the participants had periodontal disease. Bivariate analysis revealed high odds for male sex (OR = 1.31, 95% C.I = 0.44–4.84, p = 0.65) and periodontitis (OR = 3.65, 95% C.I = 0.79–26.15, p = 0.13) but low odds for a high BMI (OR = 0.45. 95% C.I = 0.07–1.67, p = 0.30). Multivariate regression revealed a significant association between insulin resistance and periodontitis. (AOR = 3.52, 95% C.I = 1.19–1.83, p = 0.03).

**Conclusion:**

Insulin resistance is highly prevalent in patients with diabetes mellitus in Uganda and is associated with periodontitis and low body weight.

## Introduction

Despite the different clinical and public health interventions, the prevalence of diabetes mellitus keeps on rising, with an estimated about 463 million people reported to be affected globally by 2019 [1]. In Uganda, different studies have reported a higher prevalence of diabetes mellitus among the adult population [2, 3] .

Type 2 diabetes mellitus, primarily results from insulin resistance, an impaired target cell response to the biological effects of insulin [4]. Though a predominant feature of type 2 diabetes mellitus, insulin resistance has also been reported in patients with type 1 diabetes mellitus as evidenced by failure to restore whole body and hepatic insulin sensitivity following islet transplantation and intensive insulin treatment in these patients [5]. Insulin resistance is one of the causes of chronic hyperglycemia in patients with diabetes mellitus [6]. This prolonged hyperglycemia is responsible for the initiation and progression of diabetic complications [7]. Overweight and obesity have been identified as important risk factors for development of insulin resistance [8]. Obesity has been reported to be associated with chronic inflammation and lipotoxicity [9]. This chronic inflammation increases the risk of developing type two diabetes mellitus and insulin resistance [10]. Tomuyuki et al., [11] also identified aging to be associated with an increased risk of developing insulin resistance. Patients with advanced age have been reported to have a defect in insulin receptor action which may cause insulin resistance and carbohydrate intolerance in the elderly [12]. Furthermore, elderly individuals are more likely to be obese and this, coupled with decreased physical activity predispose such individuals to development of diabetes mellitus and insulin resistance [13]. Another important risk factor for diabetes mellitus and insulin resistance is periodontal disease [14]. About 42.4% of patients with diabetes mellitus have been reported to have periodontal disease [15] .The pro-inflammatory immunomodulators and cytokines released as a result of periodontal disease may amplify insulin resistance [16, 17]. There are different methods of determining insulin resistance. The reference method is the hyperinsuleamic euglyceamic clamp method (HEC), an in vivo method that requires constant insulin infusion and monitoring the hypoglycemic effects of insulin [18]. However, simpler methods like the Homeostasis Model Assessment for Insulin Resistance (HOMA -IR) and Triglyceride Glucose Index (TyG) can also be used to give a good picture of the degree of insulin resistance in the body [19, 20]. Although the HOMA-IR is a good indicator of insulin sensitivity, it is expensive and not readily available in most laboratories. On the other hand, the Triglyceride Glucose Index is simpler and cheaper [21]. It has also been proven to be a strong predictor for insulin resistance in participants with and without diabetes mellitus [22]. In Uganda, a large portion of patients living with diabetes mellitus have poor glycemic control [23] Unfortunately, there is scanty information about the association between periodontitis and insulin resistance. This study set out to determine the association between periodontitis and insulin resistance in adult patients with diabetes mellitus in Uganda using the Triglyceride glucose index.

## Methods

### Study design

This was a cross sectional study conducted between December 2021 and February 2022 at the Diabetes Out- patient Clinic in Kiruddu National Referral Hospital located in Kampala, Uganda.

### Study participants

The study included 223 patients, aged 18 years and above, with a confirmed diagnosis of diabetes mellitus [24]. Participants who were pregnant, on hormonal contraceptive therapy, very sick or on broad spectrum antibiotics were excluded from the study. Written informed consent was obtained from the participants prior to enrolment in the study.

### Sample size estimation

The study sample size was calculated using a formula for finite population assuming 75% proportion of participants with insulin resistance, 90% confidence interval and 5% margin of error. A 8% was added to cater for loss to follow up [25]. The study included 223 participants. Consecutive sampling method was used to obtain the required number of participants who met the inclusion criteria.

### Measurements

An oral examination was carried out by a trained dental surgeon to determine the community periodontal index (CPI). Periodontal status was recorded with the aid of a periodontal probe (Koushen, China) and recorded using the World Health Organization Oral Health Assessment Form For Adults, 2013. The results were interpreted as follows: 0 normal; 1 bleeding; 2 calculus; 3 pocket depth < 4 mm and 4 pocket depth > 4 mm. Participants with CPI values of 0 to 2 were reported as having no periodontitis while those with CPI values of 3 and 4 were reported as having periodontitis. The weight (kg) of the participants was measured using a calibrated weighing scale (KINLEE, China) with the participants wearing light clothing and the height (cm) using a standardised measuring tape (Boyloo, China) with the participants barefooted [26]. These were used to calculate the body mass index (kg/m^2^). The participants were grouped into to depending on their BMI. The normal BMI (BMI ≤ 24.9) and high BMI (BMI > 24.9). Approximately 4mls of venous blood was collected following eight hours of fasting to determine the serum triglycerides (mg/dL), fasting blood sugar levels (mg/dL) and glycated hemoglobin (HbAIc, %) using the Cobas Integra 400 plus. Insulin resistance was determined using the Triglyceride Glucose index (TyG), calculated as In [triglycerides (mg/dL) X fasting blood glucose (mg/dL)/2] [27]. Participants with a TyG index greater than 8.5 were considered to be insulin resistant [28]. The income status was determined using the monthly income in Uganda shillings. Participants earning less than four hundred thousand shillings were classified under low-income status while those earning more than four hundred thousand Uganda shillings were classified under high economic status. Smoking status was determined based on history of active smoking while frequency of tooth cleaning was determined on how often participants cleaned their teeth daily.

### Data analysis

Data were entered in Microsoft excel (version 16.75.2), cleaned and exported to R (version 4.10) for analysis. Descriptive statistics were used to summarize the data. Logistic regression analysis was performed to determine the association between the different variables. The independent variables were age, BMI and periodontitis while the dependent variable was insulin resistance. All variables were retained in the Multivariate model because of their biological plausibility. The results were presented as Odds ratio with a 95% confidence interval. P-values less or equal to 0.05 were reported to be statistically significant.

## Results

### Descriptive results

Of the 223 participants, 70% were females and the average age was 48.5 (SD +/ 11.1) years. (Table 1). The mean Hb1Ac (%) was 8.96 (SD+/- 2.63) and the mean BM1 (kg/m^2^) was 29.6 (SD+/-5.82) with 30% of the participants being underweight or normal (BMI ≤ 24.9) and the rest being overweight or obese (BMI > 24.9). The majority of participants had attained formal education. About 55.6% of the study participants were on oral hypoglycemics, 12.1% on insulin and 32.3% on combination therapy. Only 17.9% of the participants were earning more than Uganda shillings 400,000= (about United States Dollars 108) per month. The mean triglyceride glucose index was 9.48 (SD+/- 0.675) mg/dL. About 93% of the study participants had insulin resistance while 58.3% of the participants had periodontitis (Table 1).

**Table 1:** A table of descriptive characteristics of study participants

Attribute	Overall Freq (%) (N = 223)
**Sex**	
Female	156 (70.0)
Male	67 (30.0)
**Age**	
Mean (SD)	48.5 (11.1)
**Education status**	
No formal education	29 (13.0)
Primary	107 (48.0)
Secondary	69 (30.9)
Tertiary	18 (8.1)
**Income status (pm)**	
Low (< 400,000UgShs or 108 USD)	183 (82.1)
High (> 400,000UgShs or 108 USD)	40 (17.9)
**Frequency of teeth cleaning**	
Inadequate (less than twice a day)	89 (39.9)
Adequate (more than twice a day)	134 (60.1)
**Mode of teeth cleaning**	
Inadequate (didn’t use any conventional teeth cleaning methods)	12 (5.3)
Adequate (used a conventional teeth cleaning method)	211 (94.6)
**History of active smoking**	
No	213 (95.5)
Yes	10 (4.5)
**Type of diabetic medication**	
Oral	124 (55.6)
Insulin	27 (12.1)
Both	72 (32.3)
**BMI** (Kg/m^2^)	
Mean (SD)	29.6 (5.82)
**TyG index**	
≤8.5	16 (7.2)
>8.5	207 (92.8)
**HbA1c (%)**	
Mean (SD)	8.97 (2.63)
**Periodontitis**	
Absent	93 (41.7%)
Present	130 (58.3%)

### Factors associated with insulin resistance

Participants were assessed for the association between insulin resistance and selected variables namely age, BMI and periodontitis. Bivariate analysis revealed high odds for male sex (OR = 1.31, 95% C.I = 0.44–4.84, p = 0.65) and periodontitis (OR = 3.65, 95% C.I = 0.79–26.15, p = 0.13) but low odds for high BMI (OR = 0.45. 95% C.I = 0.07–1.67, p = 0.30). Logistic regression revealed a significant association between insulin resistance and periodontitis. (AOR = 3.52, 95% C.I = 1.19–11.83, p = 0.03) (Table 2).

**Table 2:** Logistic regression analysis of factors associated with insulin resistance

Dependent: Triglyceride glucose index	Attributes	≤ 8.5(normal)	> 8.5 (high)	OR (bivariable)	AOR (multivariable)
Age	Mean (SD)	49.8 (13.2)	48.4 (10.9)	0.99 (0.94–1.04, p = 0.63)	0.98 (0.93–1.03, p = 0.43)
Sex	Female	12 (7.7)	144 (92.3)	-	-
	Male	4 (6.0)	63 (94.0)	1.31 (0.44–4.84, p = 0.65)	1.01 (0.32–3.84, p = 0.1)
BMI(kg/m^2^)	Normal (≤ 24.9)	2 (3.8)	50 (96.2)	-	-
	High (> 24.9)	14 (8.2)	157 (91.8)	0.45 (0.07–1.68, p = 0.30)	0.49 (0.07–1.94, p = 0.38)
Periodontitis	Absent	11 (11.8)	82 (88.2)	-	-
	Present	5 (3.8)	125 (96.2)	3.35 (1.17–10.97, p = 0.03)	3.52 (1.19–11.83, p = 0.03)

## Discussion

This study set out to determine the prevalence of insulin resistance and its association with periodontitis in participants with diabetes mellitus attending the medical outpatient clinic at a National Referral hospital in Uganda. The prevalence of insulin resistance among the study participants was 92.8% and this was significantly associated with periodontitis.

This study reports a high prevalence of insulin resistance. The prevalence of insulin resistance was more than twice that reported by Bermudez et al. [29]. However, that particular study looked at the general population while our study focused on only patients with diabetes mellitus. A large percentage of patients with diabetes mellitus in Uganda have type 2 diabetes mellitus, which is primarily associated with insulin resistance [3, 30]. This is further complicated by the inadequate and inappropriate anti-diabetic medications that has been reported amongst patients with type two diabetes mellitus [31]. The high rates may also be due to that fact that these are referral hospitals so patients who have severe forms or poorly controlled diabetes mellitus are seen.

Participants with periodontitis were three and a half times more likely to have insulin resistance than those without periodontitis. Periodontitis, being an inflammatory condition caused by gram negative bacteria, leads to a spill of inflammatory cytokines into the blood stream leading to systemic inflammation [32]. Some of these proinflammatory cytokines may amplify insulin resistance [33]. Indeed In -Seok Song et al., reported a positive association between severe periodontitis and insulin resistance even in patients with normal waist circumference [34]. Shravani Babladi et al., further demonstrated a progressive decline in insulin resistance following periodontal treatment [35]. Further studies need to be conducted to determine whether periodontal treatment is associated with a reduction in insulin resistance in the Uganda diabetic population.

Females in this study were two times more likely to have insulin resistance than males. This finding is consistent with Ramin Alemzadeh and Jessica Kichler who reported an association between insulin resistance and the female sex [36]. However, Greenhill reported that women are less susceptible to insulin resistance before menopause but the risk is similar to men after menopause, the reason for this being the protective effect of female sex hormones [37]. Our findings may be due to the older age of the study participants.

There was a negative association between a higher BMI and insulin resistance in this study. Participants who had a higher BMI had a 45% reduced chance of having insulin resistance than those with a lower BMI. This finding is contrary to Sun Min Lim et al., who reported a positive association between BMI and insulin resistance [38]. Obesity has been linked with chronic inflammation and insulin resistance [39]. However, our findings suggest a reduced risk of insulin resistance in participants who are overweight or obese. However, Umesh et al. reported insulin resistance in patients with lower BMI citing different pathways involved in this mechanism [40]. Mohammed et al., also reported insulin resistance in the non-obese participants, though his study identified an increased risk with increased BMI [41]. Though many studies grade obesity using BMI, many clinically non-obese individuals may actually be metabolically obese since the mechanisms of insulin resistance in the obese and the non-obese individuals is identical [42]. The reverse may be true where clinically obese individuals may be metabolically non- obese. This may explain why the otherwise obese participants in our study had a lower chance of having insulin resistance.

The results from this study indicate a statistically significant association between periodontitis and insulin resistance in adult patients with diabetes mellitus in Uganda. Further studies need to be conducted to determine whether periodontal treatment is associated with decreases insulin resistance in patients with diabetes mellitus in Uganda.

### Limitations of the study

This study was limited by a COVID-19 and Ebola outbreaks which greatly hindered patient recruitment.

This study was also conducted in a National Referral Hospital where most of the patients with severe disease are referred to hence these results may not be generalized to the whole population.

One of the independent variables was body mass index (BMI). BMI poorly correlates with visceral adiposity compared with waist circumference and waist -hip ratio [43].

## Data Availability

The data sets generated and analyzed during the current study will be available from the corresponding author on request.
